# Investigation of acute febrile illness outbreak- Asyaita and Dupti districts, Afar Region, Ethiopia, February 2011

**DOI:** 10.1186/1742-4690-9-S1-P46

**Published:** 2012-05-25

**Authors:** Abyot Bekele Woyessa, Worknesh Ayele, Abdi Ahimed, A Nega

**Affiliations:** 1 Ethiopian Health and Nutrition Research Institute, Ethiopia, Addis Ababa, Ethiopia

## Introduction

Acute Febrile Illnesses (AFIs) due to different etiologic agents are the most common causes of morbidity and mortality in developing tropical and subtropical countries. Afar region reported unidentified AFI outbreak on 10-Aug-2011. We investigated to identify etiologic agent, risk factors and to recommend prevention and control measures.

## Methods

Unmatched case control study was employed. Study subjects (57 cases and 57 controls) were obtained and interviewed. Cases were defined as any person with fever ≥ 38.3C°, headache, pains in joints, muscles and back, anorexia and weakness. Medical records were reviewed and suspected AFI cases were identified from 07-Aug-2011 to 11-Sep-2011in Asyaita and Dupti districts. Active cases were searched house to house. Blood-samples, blood-serums and throat-swabs were collected and analyzed for heamoparasites, bacterial-pathogens, hemorrhagic fevers and respiratory viruses at national and CDC Kenya laboratories. Environmental scanning was performed. Odd-Ratio (OR) in 95%Confidence-Interval (CI) was calculated using Epi-Info version-3.5.1.

## Results

A total of 12816 suspected AFI cases with no death were identified. Of the cases 9107(71%) were male and 3709 (29%) were female. Attack-Rate (AR) was 8.7% (11.5% in male and 5.4% in female) and 13.8% among 15-44 age-groups. On bivariate analysis factors associated with illness were living with sick family member (OR: 2.8; 95%CI: 1.3-6.2), contact with patient (OR: 3.8; 95%CI: 1.5-9.6) and drinking deep-well water (OR: 2.7; 95% CI: 1.2-5.8). However, on multivariate analysis only having contact with patient (OR: 4.1; 95%CI: 1.5-11.3) was associated with illness. Twenty-five specimens were tested negative for malaria parasites, Salmonella species, brucella species, dengue fever, yellow fever and rift valley fever.

## Conclusions

An outbreak of suspected AFI occurred in 2 districts affecting primarily males and older age. Having contact history with patient was risk factor to contract the illness. Etiologic agent and source of the outbreak was not identified yet. Large-scale investigation is recommended.

